# Supplementary material for: Exosomal CD40, CD25, and Serum CA19-9 as Combinatory Novel Liquid Biopsy Biomarker for the Diagnosis and Prognosis of Patients with Pancreatic Ductal Adenocarcinoma
Source: Int J Mol Sci. 2025 Feb 11;26(4):1500. doi: 10.3390/ijms26041500 (PMC11854914; doi:10.3390/ijms26041500)
Supplement: Supplementary file 1 [file ijms-26-01500-s001.zip › ijms-3477735-supplementary.pdf]

Supplementary

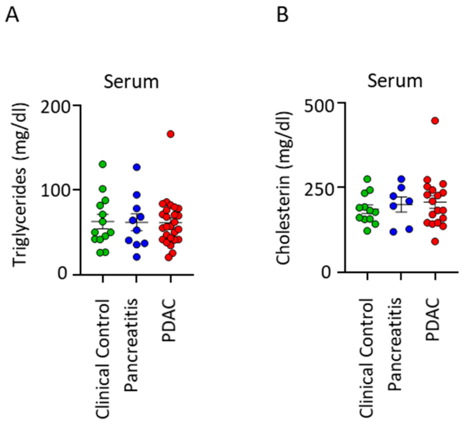

Supplementary Figure S1: - Detection of lipid levels in clinical control, Pancreatitis and PDAC patients. A. Triglycerides level in clinical control, pancreatitis and PDAC patients. B. Cholesterin levels in clinical control, pancreatitis and PDAC patients.

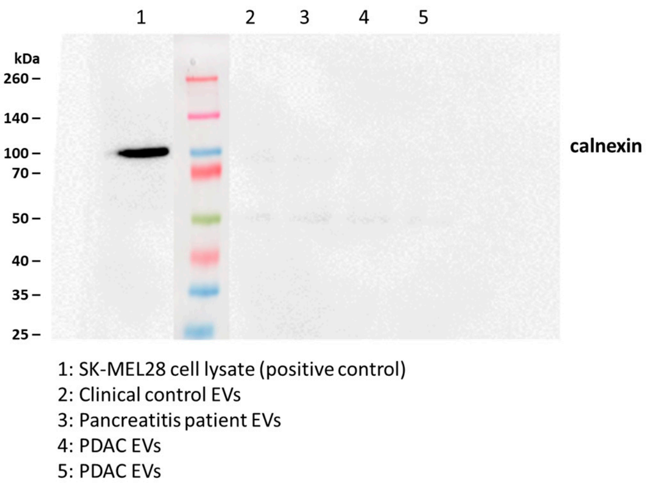

Supplementary Figure S2. Validation of isolated exosomes. Western blot denoting the ladder with molecular weight, negative exosome marker (Calnexin) and the numeric of the lane.

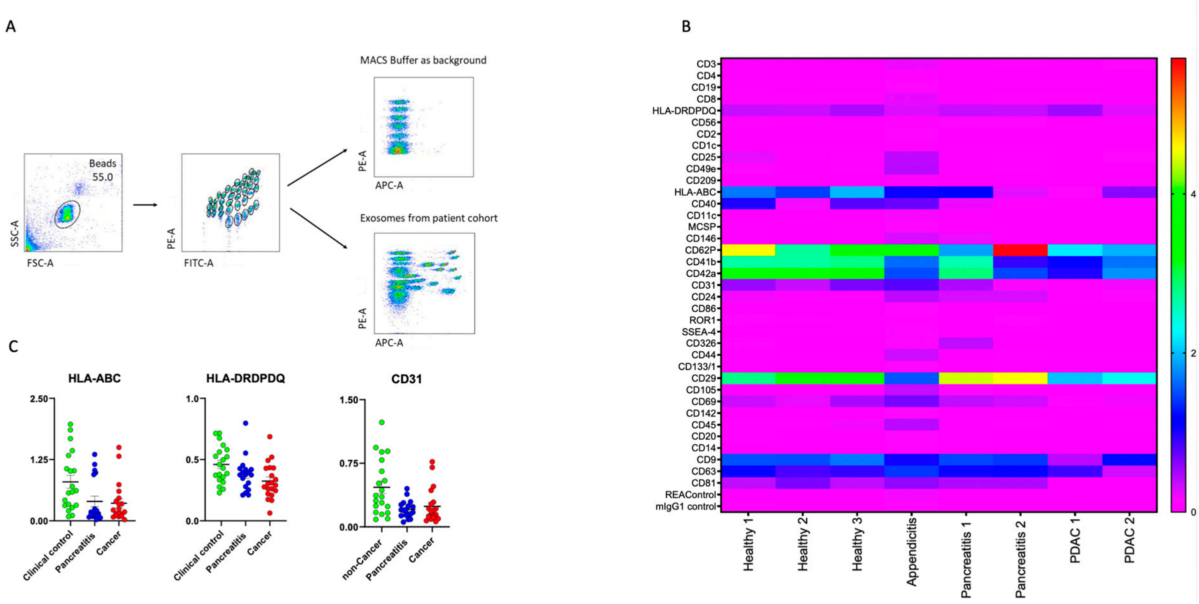

Supplementary Figure S3: Biomarkers to distinguish patients with pancreatic ductal adenocarcinoma (PDAC) from those with pancreatitis and other clinical control subjects. (A) Gating strategy to characterize exosomes from our patient cohort. (B) Heat map denoting the expression level of 37 exosomal biomarkers and 2 controls in our patient cohort. (C) Expression of exosomal markers in clinical controls, pancreatitis and PDAC patients.

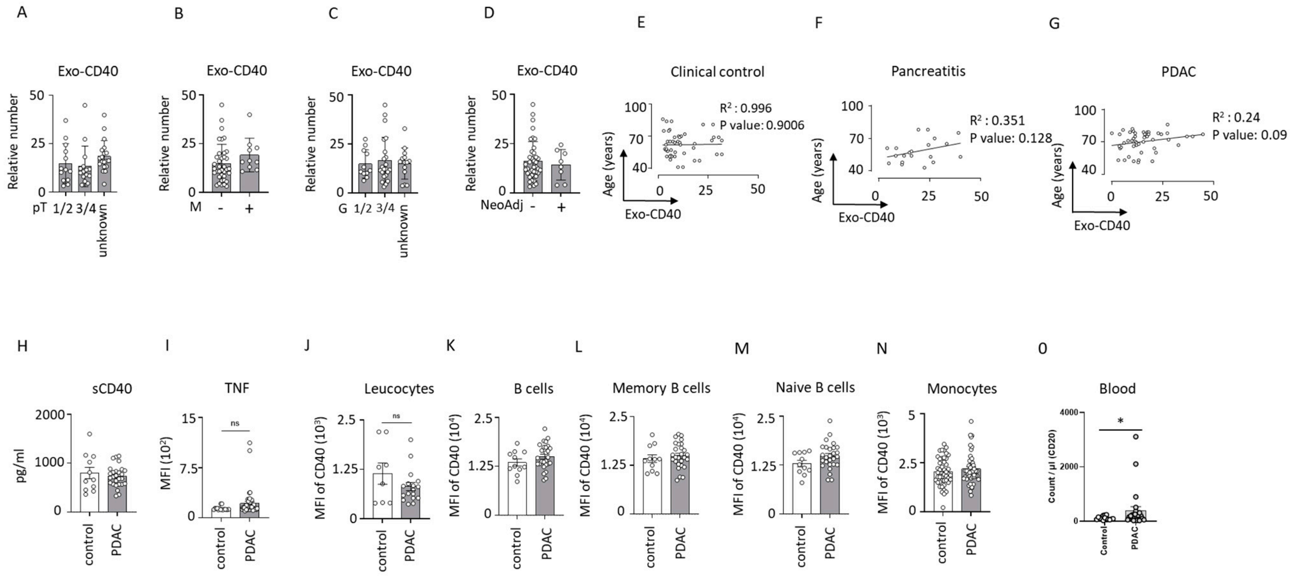

Supplementary Figure S4: - exo-CD40 as a novel marker to discriminate PDAC patients from non-cancerous patients. A-D) Clinical correlation of PDAC patients according to pathological examination. E-G) Correlation of exo-CD40 and age of individuals from PDAC, non-cancer and pancreatitis cohort. (H) soluble CD40 levels in clinical controls and PDAC patients. (I) TNF levels of control and PDAC patients. (J-N)) Expression of CD40 on

leucocytes, B cells, memory B cells, naive B cells and monocytes. (O). B cells absolute count in the peripheral blood of clinical control and PDAC patients.

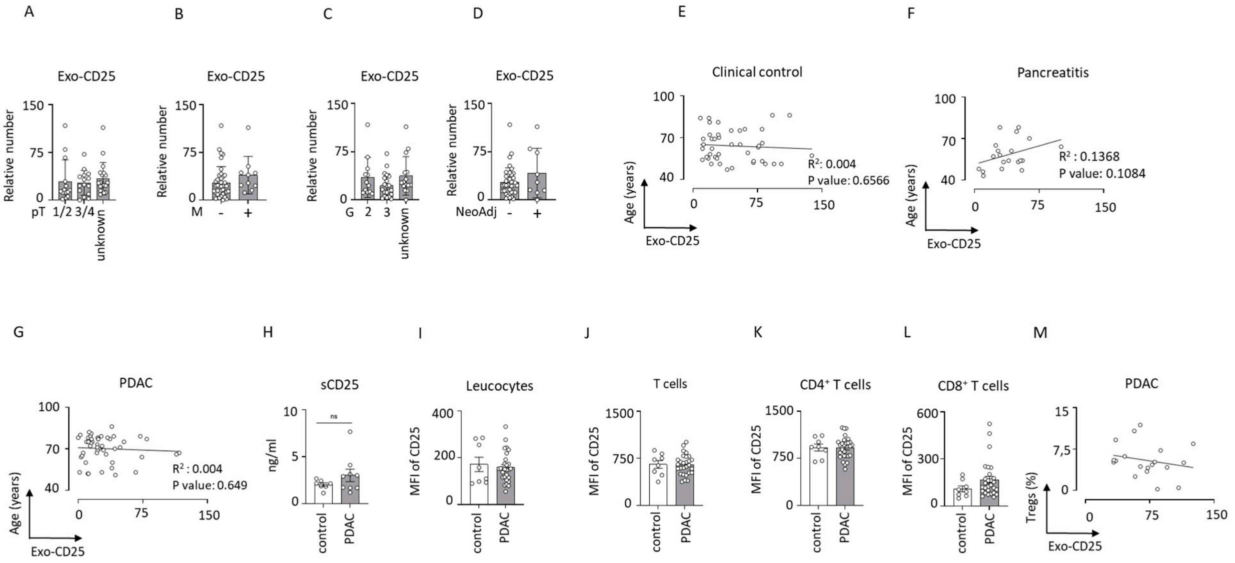

Supplementary Figure S5: - exo-CD25 as a novel marker to discriminate PDAC patients from pancreatitis and clinical control individuals. (A-D) Clinical correlation of PDAC patients according to pathological examination. (E-G) Correlation of exo-CD25 and age of individuals from PDAC, non-cancer and pancreatitis cohort. (H) soluble CD25 levels in clinical controls and PDAC patients. (I-L) Expression of CD25 on leucocytes, T cells, CD4+ T cells and CD8+ T cells. (M) Correlation of exo-CD25 and frequencies of circulating Tregs of PDAC patients.

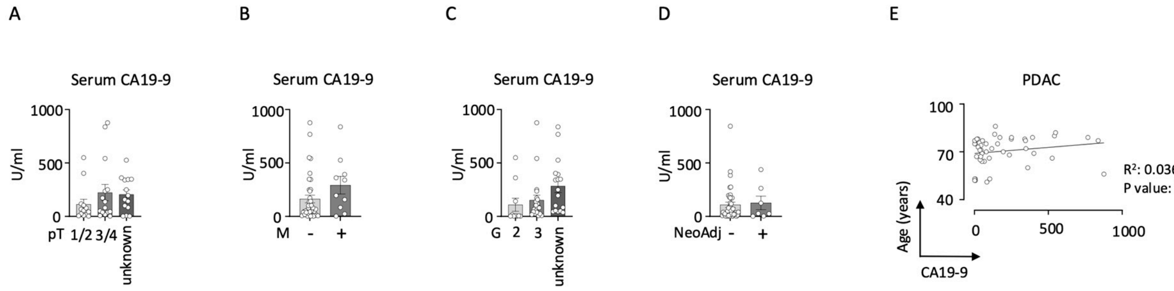

Supplementary Figure S6: - Serum CA19-9 to discriminate PDAC patients from pancreatitis and clinical control individuals. (A-D) Clinical correlation of PDAC patients according to pathological examination. E) Correlation of exo-CD40 and age of individuals from PDAC.

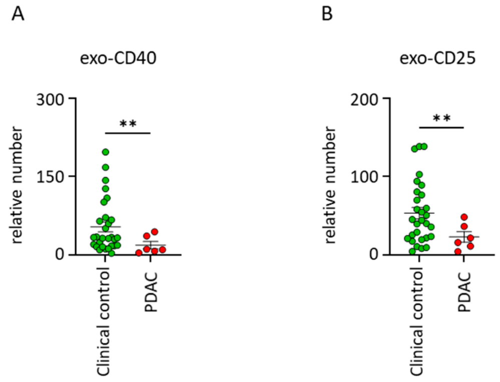

Supplementary Figure S7: - Plot denoting the expression of exo-CD40 and exo-CD25 of clinical control patients. **A**. exo-CD40 levels of PDAC patients with low CA19-9 plotted against clinical control patients. **B**. exo-CD25 levels of PDAC patients with low CA19-9 plotted against clinical control patients.

Table S1: Clinic-pathological characteristics of the PDAC patient cohort grouped according to the median of exo-CD40.

**CD40 PDAC**		**Low**	**High**	**p-Value**
Number		23	25	
Mean Age (in years [range])		62.2 (51-82)	69.8 (52-86)	0.1938
Sex	Female	11	12	1.0
	Male	12	13	
pT/ypT	1	2	0	**0.02**
	2	6	6	
	3	9	4	
	4	2	0	
	Unknown/non-resectable	4	15	
pN/ypN	0	6	6	0.05
	1	6	4	
	2	8	3	
	Unknown/Inoperable	3	12	
Lymphatic invasion	L0	12	8	0.05
	L1	6	3	
	Unknown/Inoperable	5	14	
Venous invasion	V0	18	10	**0.02**
	V1	0	2	
	Unknown/Inoperable	5	13	
Perineural invasion	Pn0	7	3	**0.03**
	Pn1	12	9	
	Unknown/Inoperable	4	13	
R-status	0	16	10	**0.02**
	1	3	1	
	2 (inoperable)	4	14	
Distant Metastasis	M0	20	18	0.20
	M1	3	7	
UICC stage	I	3	2	**0.04**
	II	7	5	
	III	8	2	

	IV	3	7	
	Unknown	2	9	
Neoadjuvant treatment	Yes	3	5	0.52
	No	20	20	
Mean Preoperative CA19-9 (in u/mL [range])		134.6 (24.1-541.8)	182.7 (6.9-392.9)	0.3791

Table S2: Clinic-pathological characteristics of the PDAC patient cohort grouped according to the median exo-CD25.

CD25 PDAC		Low	High	p-Value
Number		23	25	
Mean Age (in years [range])		67.4 (52-82)	69.2 (51-86)	0.9472
Sex	Female	12	11	0.54
	Male	11	14	
pT	1	1	1	0.23
	2	9	3	
	3	6	7	
	4	1	1	
	Unknown/Inoperable	6	13	
pN	0	4	8	0.13
	1	6	4	
	2	8	3	
	Unknown/Inoperable	5	10	
Lymphatic invasion	L0	9	11	0.02
	L1	8	1	
	Unknown/Inoperable	6	13	
Venous invasion	V0	17	11	0.07
	V1	0	2	
	Unknown/Inoperable	6	12	
Perineural invasion	Pn0	5	5	0.14
	Pn1	13	8	
	Unknown/Inoperable	5	12	
R-status	0	15	11	0.23
	1	2	2	
	2 (inoperable)	6	12	
Distant Metastasis	M0	21	17	0.05
	M1	2	8	
UICC stage	I	2	3	0.10
	II	5	7	
	III	8	2	
	IV	2	8	
	Unknown	6	5	
Neoadjuvant treatment	Yes	3	5	0.52
	No	20	20	
Mean Preoperative CA19-9 (in u/mL [range])		96.3 (8.3-541.8)	201.3(6.9-526.5)	0.0573

Table S3: Clinic-pathological characteristics of the PDAC patient cohort grouped according to the median CA19-9 level on plasma.

**CA19-9 PDAC**		**Low**	**High**	**p-Value**
Number		23	25	
Mean Age (in years [range])		66.7 (52-78)	70.0 (51-86)	0.3105
Sex	Female	11	12	0.1
	Male	12	13	
pT	1	1	1	0.12
	2	9	3	
	3	7	6	
	4	1	1	
	Unknown/Inoperable	5	14	
pN	0	8	3	**0.03**
	1	5	5	
	2	6	3	
	Unknown/Inoperable	4	14	
Lymphatic invasion	L0	12	8	0.12
	L1	5	4	
	Unknown/Inoperable	5	13	
Venous invasion	V0	17	11	0.09
	V1	1	1	
	Unknown/Inoperable	5	13	
Perineural invasion	Pn0	6	4	**0.04**
	Pn1	13	8	
	Unknown/Inoperable	4	13	
R-status	0	14	12	0.21
	1	3	1	
	2 (inoperable)	6	12	
Distant Metastasis	M0	21	17	0.05
	M1	2	8	
UICC stage	I	3	1	**0.03**
	II	5	3	
	III	8	2	
	IV	2	8	
	Unknown	5	11	
Neoadjuvant treatment	Yes	3	5	0.52
	No	20	20	

Table S4: Median value of the relative numbers of the screened biomarkers.

**Biomarkers**	**Clinical control**	**Pancreatitis**	**PDAC**
Exos-CD326	2,4807378	2,3745984	3,14243582
Exos-CD3	10,5476042	11,1126534	8,82071355
Exos-CD4	1,37864967	1,08042284	0,71593049
Exos-CD19	1,56607939	0,91710079	1,15461668
Exos-CD8	8,86710679	7,38289787	7,91540649

Exos-HLA-DRDPDQ	37,8042635	37,4292049	38,270212
Exos-CD56	12,1623935	13,4169561	12,8542984
Exos-CD105	3,39901721	2,73030251	3,53715392
Exos-CD2	1,79143956	1,39442701	1,02711979
Exos-CD1c	1,73521167	1,29184419	1,44053957
Exos-CD25	39,874475	39,6630044	22,6413378
Exos-CD49e	7,22891566	5,00510896	4,57061608
Exos-ROR1	6,75097795	3,4808669	3,47380248
Exos-CD209	1,33228415	0,73950091	0,85414608
Exos-SSEA-4	1,86321163	1,8945041	1,71108771
Exos-HLA-ABC	35,2694305	14,7144233	14,2805913
Exos-CD40	32,6182666	12,8923979	13,1440079
Exos-CD62P	351,63762	368,968474	302,596758
Exos-CD11c	1,1299131	0,71510001	0,58815061
Exos-MCSP	1,59559413	0,67724092	1,31558168
Exos-CD146	2,24773692	2,69324407	2,76628024
Exos-CD41b	219,456978	174,726309	150,400908
Exos-CD42a	181,694345	119,815119	88,6571934
Exos-CD24	12,6530459	10,817631	10,2086148
Exos-CD86	2,59053336	1,49653848	1,98439012
Exos-CD44	7,29726353	4,89018971	5,53037391
Exos-CD133/1	2,80110763	1,72550213	2,63198034
Exos-CD29	262,964148	223,116187	241,449713
Exos-CD69	28,8890023	24,6785174	23,4508405
Exos-CD142	1,39469257	0,71169438	1,00025744
Exos-CD45	15,8001845	9,9208109	7,16842581
Exos-CD31	32,4303579	19,4966853	17,5776197
Exos-CD20	2,05887431	1,07273281	1,3569736
Exos-CD14	2,24599931	1,86252772	2,00336285
Exos-CD9	145,746663	141,482996	145,728204
Exos-CD63	93,4353504	92,1560531	81,1196428
Exos-CD81	51,7464325	61,2840054	53,9307777
REA control	0,03284558	0,01365119	0,02249699
mIgG1 control	0,02537649	0,00487445	0,01765243
